# Multi-Particle Tracking in Complex Plasmas Using a Simplified and Compact U-Net

**DOI:** 10.3390/jimaging10020040

**Published:** 2024-01-31

**Authors:** Niklas Dormagen, Max Klein, Andreas S. Schmitz, Markus H. Thoma, Mike Schwarz

**Affiliations:** 1 NanoP, TH Mittelhessen University of Applied Sciences, D 35392 Giessen, Germany; max.klein@ei.thm.de (M.K.); mike.schwarz@ei.thm.de (M.S.); 2 I. Physikalisches Institut, Justus Liebig Universitat Giessen, D 35392 Giessen, Germany; andreas.s.schmitz@physik.uni-giessen.de (A.S.S.);

**Keywords:** dusty plasma, image analysis, neural networks, U-Net, paricle tracking

## Abstract

Detecting micron-sized particles is an essential task for the analysis of complex plasmas because a large part of the analysis is based on the initially detected positions of the particles. Accordingly, high accuracy in particle detection is desirable. Previous studies have shown that machine learning algorithms have made great progress and outperformed classical approaches. This work presents an approach for tracking micron-sized particles in a dense cloud of particles in a dusty plasma at Plasmakristall-Experiment 4 using a U-Net. The U-net is a convolutional network architecture for the fast and precise segmentation of images that was developed at the Computer Science Department of the University of Freiburg. The U-Net architecture, with its intricate design and skip connections, has been a powerhouse in achieving precise object delineation. However, as experiments are to be conducted in resource-constrained environments, such as parabolic flights, preferably with real-time applications, there is growing interest in exploring less complex U-net architectures that balance efficiency and effectiveness. We compare the full-size neural network, three optimized neural networks, the well-known StarDist and trackpy, in terms of accuracy in artificial data analysis. Finally, we determine which of the compact U-net architectures provides the best balance between efficiency and effectiveness. We also apply the full-size neural network and the the most effective compact network to the data of the PK-4 experiment. The experimental data were generated under laboratory conditions.

## 1. Introduction

Fundamentally, plasmas are ionized gases where electrons can move freely within the gas [1]. The density of positive and negative charge carriers in plasmas is approximately equal. When particles are introduced into a low-temperature and low-pressure discharge plasma, it is termed as a dusty or complex plasma. These plasmas, in addition to electrons, ions, and neutral gas atoms, also include micron-sized particles [1]. The high mobility of electrons in low-temperature plasma leads to the negative charging of micro-particles. They collect charged plasma particles. The structures and dynamics of large micro-particle systems, containing up to $10^{6}$ particles, can be easily observed with laser illumination due to the significant interparticle distance, typically exceeding 100 μm [1,2]. This creates a dilute and transparent particle system that can be effectively analyzed using cameras. The PK-4 experiment (“Plasmakristallexperiment 4”) is designed specifically to investigate complex plasma in a DC discharge that occurs in an elongated glass tube. A detailed description of PK-4 can be found in the reference [3]. This work is an extension of previous work [4] originally reported in the 30th International Conference on Mixed Design of Integrated Circuits and Systems—MIXDES 2023. To analyze particle behavior in the plasma, their positions must first be determined. Since each image contains several particles, the method should be able to track multiple particles in one image.

Approaches to particle detection ranged from local-maxima finding to linear filtering, linear and nonlinear model fitting, and centroid estimation schemes [5]. In the previously employed method, image preparation involves utilizing a bandpass and a threshold value. The selection of an optimal threshold is crucial for images containing features (particles) and background (noise) in image processing [6]. An optimal threshold is crucial, as emphasized in studies like Sezgin and Sankur [7]. Given the fundamental focus of Mohr et al. (2019)’s work on complex plasmas [6], we chose to employ Otsu’s method [8] as a thresholding technique in line with their research. While other thresholding techniques are available (for an overview, see, e.g., Sezgin and Sankur [7]), our choice aligns with the approach and findings of D. Mohr et al. (2019) [6]. Their findings demonstrated that most alternative techniques often result in inaccurate binarizations. Background pixels are mistakenly identified as signals and set to white. For more details, refer to D. Mohr et al. (2019) [6]). If a particle spans multiple pixels in an image, we can determine its position with sub-pixel accuracy by calculating the weighted average position of these pixels [9]. The point determined in this manner is termed the center of mass, representing the position of a particle. The method was developed based on the open-source library “trackpy” [9]. Recently, data-driven alternatives employing deep learning have significantly enhanced quantitative digital microscopy, offering the potential for accurate and rapid image analysis. Midtvedt et al. (2021) [10] have developed a dedicated tool for particle localization, tracking, and characterization, extending to cell counting and classification. In another study, Midtvedt et al. (2022) [11] concentrated on object recognition in the realm of digital microscopy, where machine learning has made substantial progress in overcoming the limitations of classical approaches. Notably, the U-net architecture was employed. Furthermore, Huang et al. (2019) [12] have successfully applied machine learning approaches to image-based analyses of complex plasmas. These insights are intended for enhancing particle localization in complex plasmas. This work focuses on a multi-particle tracking approach using a U-net. The U-net is a convolutional network architecture designed for rapid and precise image segmentation, developed at the Computer Science Department of the University of Freiburg [13]. However, as experiments are slated for resource-constrained environments, such as parabolic flights, with a preference for real-time applications, there is a growing interest in exploring less complex U-net architectures that strike a balance between efficiency and effectiveness. Considering the limited resources, the U-net [4] presented in previous work is now slated for optimization. The primary strategy is to reduce the depth of the U-net to optimize the architecture, considering FLOPS and MACCs. A smaller number of layers results in fewer parameters and, consequently, a reduced computational load. We will design three different compact architectures. Subsequently, we will determine which of the compact U-net architectures provides the best balance between efficiency and effectiveness. Next, we will compare the full-size neural network, the optimized neural network, a well-known neural network called StarDist [14], and trackpy [9] in terms of accuracy in analyzing artificial data. StarDist is a neural network architecture designed for image segmentation tasks, particularly applied to instances where objects exhibit star-shaped structures. Developed by Schmidt et al. in 2018 [14], StarDist employs a U-net-based architecture and is trained specifically for object detection in microscopy images. It utilizes a polygonal representation of object shapes, making it well-suited for applications like cell nucleus segmentation [14]. For these analyses to be meaningful, the methods should accurately detect particle positions. We also apply the two U-net architectures to experimental data.

## 2. Experiment

The Plasmakristall 4 experiment is characterized by the fact that the plasma is ignited by a direct current discharge. This makes it possible to study complex plasmas in different plasma environments [3].

**Figure 1:**
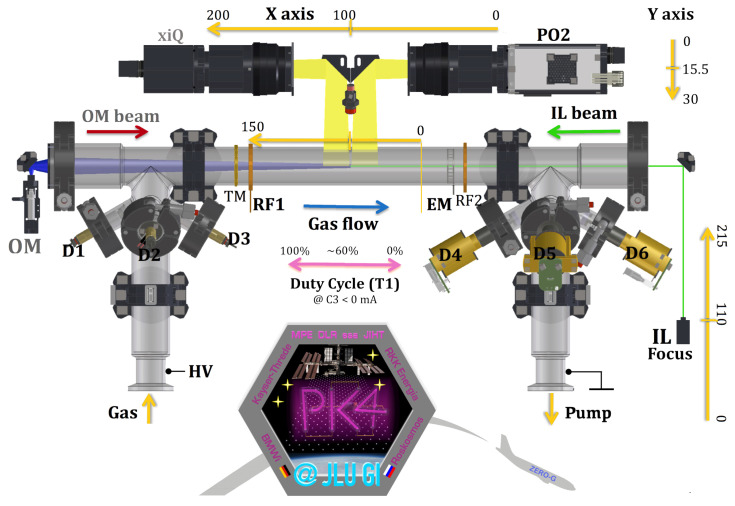
Schematic of the PK-4 plasma chamber provided by the Thoma research group of the JLU Gießen.

**Figure 2:**
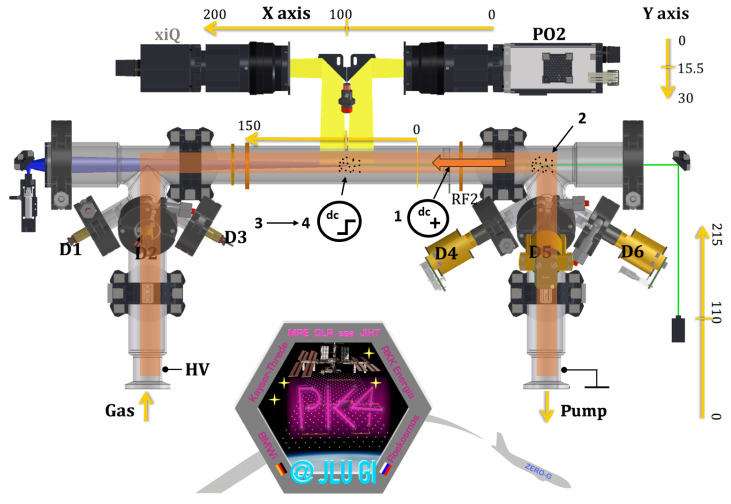
Schematic representation of DC trapping procedure. Steps: 1—ignition of the DC plasma, 2—microparticle injection, 3—microparticles arrival into the PO camera field of view, and 4—the discharge in the polarity-switching mode. The graphic was created based on the work of Pustylnik et al. [3].

A model of PK-4 has been on the International Space Station since late 2014. The details are described in ref. [3]. We will briefly outline the important components. The plasma chamber is an elongated U-shaped glass tube with a total length of 86 cm and a diameter of 3 cm. Two cameras are available for the observation of the micron-sized particles, which can be moved in longitudinal and in radial direction of the plasma chamber (x- and y-direction in Figure 1). At the ends of the glass tube, the high-voltage electrodes are mounted. The high-voltage power supply operates in DC or AC mode (polarity switching) with a frequency up to 5 kHz. The DC current can be adjusted between 0.5 and 3 mA. The electric field strength of the longitudinal DC field in the positive column of the discharge was measured using Langmuir probes on the ground, in the absence of micro-particles, to be about 2 V/cm, nearly independent of the DC current [3]. The gas, usually neon or argon with pressures between 10 and 200 Pa, is filled in by gas flow up to 10 sccm through the cylindrical electrode and can be pumped out through the other cylindrical electrode by a turbo molecular pump [2]. After igniting the plasma, the micro-particles are injected from the dispensers through ports at the side legs of the glass tube. The Particle Observation laser is used to illuminate the particles whose scattered light can be detected by the cameras. The laser emits green light with a wavelength of 532 nm and has an output power of up to 240 mW [3]. The light from the laser is fanned out to illuminate a plane perpendicular to the z-direction [3]. The scattered light from the micro-particles is recorded by a CCD camera (2 Megapixel, 35 frames per second [fps] at full resolution) and a CMOS camera with a larger field of view and a higher frame rate and resolution (xiQ MQ042MG-CM, 4 Megapixel, 90 fps at full resolution, 1 pixel corresponds to 11.4 μm) [2]. They can scan the tube 20 cm in the horizontal direction as well as 3 cm perpendicular to it. The DC mode can be used to trap and stop the micro-particles (Figure 2).

To study the particles in microgravity, experiments can be performed either on board the ISS or during parabolic flights. The I. Physikalisches Institut of the Justus-Liebig-Universität (JLU) in Gießen focuses on laboratory investigations of complex plasmas and plans/tests experiments for the ISS using the PK-4 experiment. For this purpose, the JLU has an identical model known as the Science Reference Model (SRM). This is used to plan experiments and largely automate processes. In addition, experiments are also performed with a parabolic flight model of the PK-4 in parabolic flight. This is almost identical to the SRM, but can be modified for more advanced experiments, which is not possible with the SRM. For example, other particle types can be used, or components such as cameras can be modernized. Various parabolic flight experiments have been conducted in the past with the support of the German Aerospace Center (DLR).

**Figure 3:**
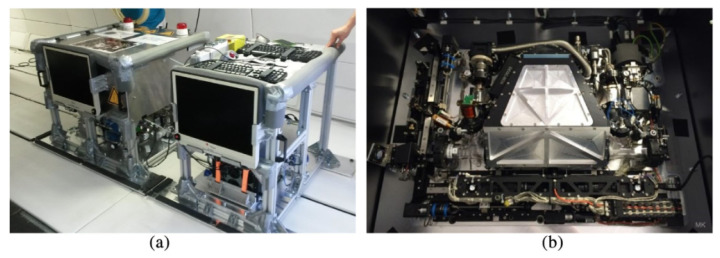
(**a**) The PK-4 parabolic flight experiment unit in the aircraft A310 Zero-G. (**b**) The integrated base plate accommodated in the left rack [2].

For the parabolic flights, the aircraft A310 ZERO-G of the company Novespace is used. Microgravity with a duration of about 22 s per parabola will be realized on three flight days in 31 parabolas each. The parabolic flight model consists of two racks (Figure 3a), one containing an integrated base plate (Figure 3b), another one containing the computers for experiment control and recording. The integrated base plate was used as the engineering model for the ISS project and is to a large content identical with the one of the flight model [2]. The micron-sized particles were injected into the plasma at the beginning of the microgravity phase of a parabola, which then move into the field of view of the cameras. Once the particles arrive into the field of view, the DC discharge is switched to the polarity-switching mode with a duty cycle of δ=0.5 [3]. After this, the micro-particles experience zero net force and are trapped [3]. Once the particles are trapped, they will be observed and photographed by the cameras. In order to perform further analyses on the plasma, the positions of the particles in the plasma have to be determined from the images. Conventional methods were used for this purpose in previous investigations. In the following, a machine learning approach will be investigated, which could potentially replace the conventional method in future investigations.

## 3. U-Net Architecture

**Figure 4:**
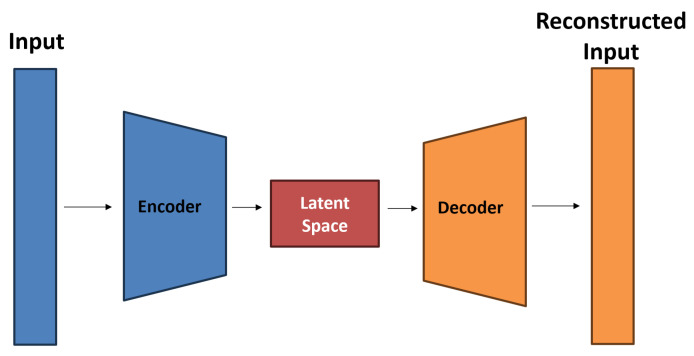
Schematic representation of an U-Net architecture.

Developed by Olaf Ronneberger, Philipp Fischer, and Thomas Brox in 2015 [13], the U-Net represents a special neural network, which is primarily designed for image segmentation tasks. It excels at partitioning an image into multiple segments or regions of interest, making it particularly suitable for tasks where precise delineation of objects or structures in images is required, such as medical image segmentation or semantic segmentation in computer vision. The U-Net architecture has a U-shaped structure with a contracting path (encoder) and an expansive path (decoder) (see Figure 4). It incorporates skip connections that preserve spatial information and allow the network to perform accurate segmentation. It has a specific focus on spatial preservation and the precise localization of objects.

The encoder is the initial part of the U-Net. It takes the input data and maps it to a lower-dimensional representation, the so called latent space. The encoder accomplishes this through a series of layers, where each layer performs mathematical transformations on the input data. These transformations pack the information step by step into a more compressed format. The U-Net’s latent space is often called the “middle layer” or “bottleneck”. While it does capture abstract representations of the input image, its primary purpose is to facilitate segmentation rather than feature extraction or dimensionality reduction. The expansive path, also known as the decoder, aims to generate a high-resolution segmentation map from the feature representations obtained in the contracting path. Unlike traditional CNN architectures, the U-Net employs a series of up-convolutions (transposed convolutions or deconvolutions) to upsample the feature maps. Moreover, the expansive path incorporates skip connections that concatenate feature maps from the contracting path. These skip connections enable the U-Net to preserve fine-grained spatial information and are essential for accurate segmentation. The U-Net’s output is segmentation masks, where each bright pixel in the mask can be assigned to a particle position.

### 3.1. Simplifying the Architecture

**Figure 5:**
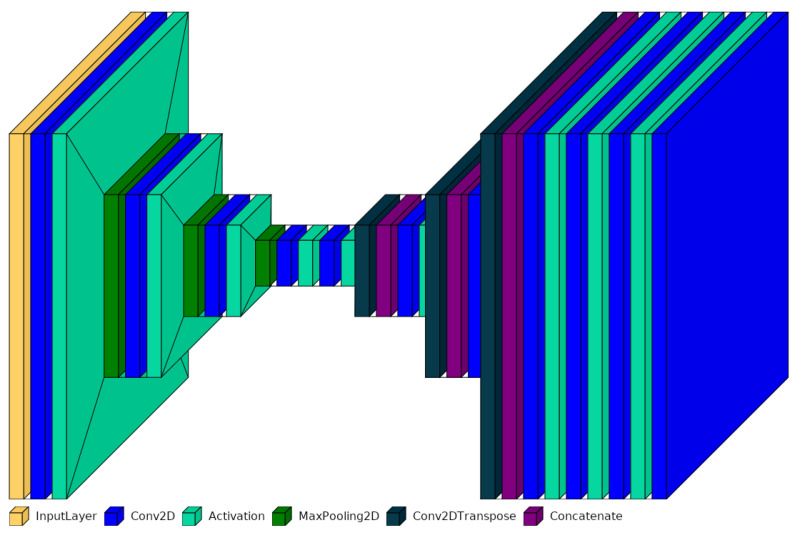
Structure of the U-Net architecture.

The traditional U-Net architecture, celebrated for its outstanding accuracy and fine-grained segmentation through contracting and expansive paths connected by skip connections, is depicted in Figure 5. This U-Net comprises numerous layers, encompassing a total of 389,521 parameters. However, this intricate structure poses a computational challenge, especially in resource-constrained settings like parabolic flights. Consequently, there is an increasing demand for models that can provide precise results without sacrificing speed or resource efficiency. Less complex U-Net architectures aim to fulfill this demand by simplifying the original design while preserving essential features for effective segmentation.

The key approach involves diminishing the depth of the U-Net. A reduced number of layers leads to fewer parameters, thereby decreasing computational demands. This modification proves especially beneficial for tasks where a shallower architecture remains capable of capturing essential features. Using lightweight convolutional blocks serves to substantially streamline the model’s complexity while preserving satisfactory segmentation quality. Diligent reduction in the number of skip connections contributes to simplifying the architecture. However, this adjustment should be executed with caution to guarantee the retention of pertinent contextual information. In accordance with this strategy, three distinct architectures were devised.

#### 3.1.1. Simplified U-Net 0

**Figure A1:**
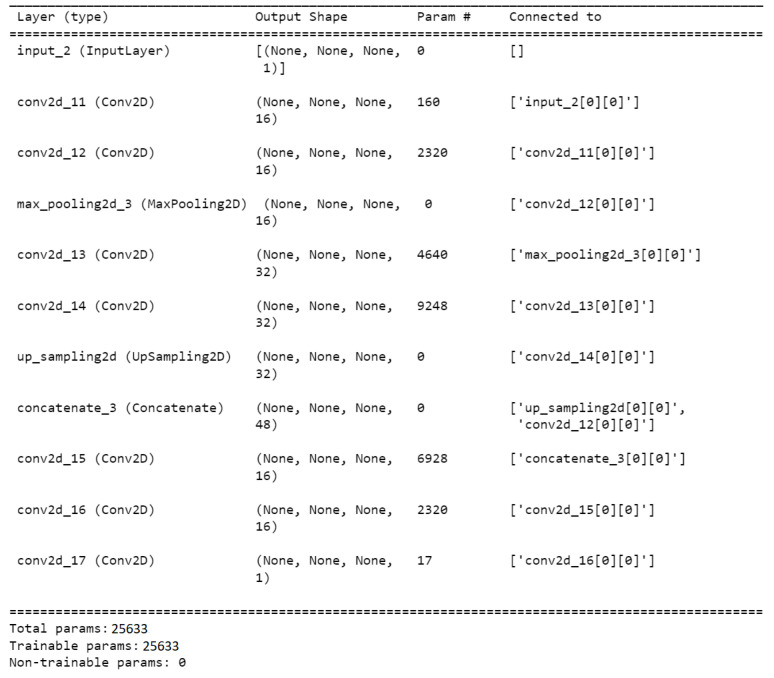
Structure of the simplified and more compact U-Net 0 architecture.

The contracting path is comprised of two convolutional layers, followes by a max-pooling layer. The initial convolutional layer has 16 filters and utilizes the ReLU activation function, with padding set to ’same’ to preserve spatial dimensions. The second convolutional layer mirrors the first, effectively doubling the number of feature maps. Max-pooling layers, with a pool size of 2 × 2, halve the spatial dimensions. The bottleneck involves two convolutional layers, each with 32 filters, followed by the ReLU activation function. This step condenses information into a latent representation. On the expansive path, the feature maps undergo upsampling and are concatenated with feature maps from the contracting path. In contrast to the original architecture, we replaced transposition operations (Conv2DTranspose, see Figure 5) with upsampling operations (UpSampling2D, see Figure A1) to enhance the network’s efficiency. Transposition operations combine trainable upsampling and convolution, providing flexibility but potentially incurring higher computational costs. On the other hand, upsampling operations perform fixed upsampling without trainable parameters, making them more efficient albeit less adaptable.

Following each upsampling layer, two convolutional layers with 16 filters and ReLU activation are applied. Concatenation merges the upsampled feature maps with the corresponding feature maps from the contracting path. This skip connection is instrumental in enabling the network to recover spatial details. Consequently, we managed to reduce the architecture to a size of 25,633 parameters (see Figure A1).

#### 3.1.2. Simplified U-Net 1

**Figure A2:**
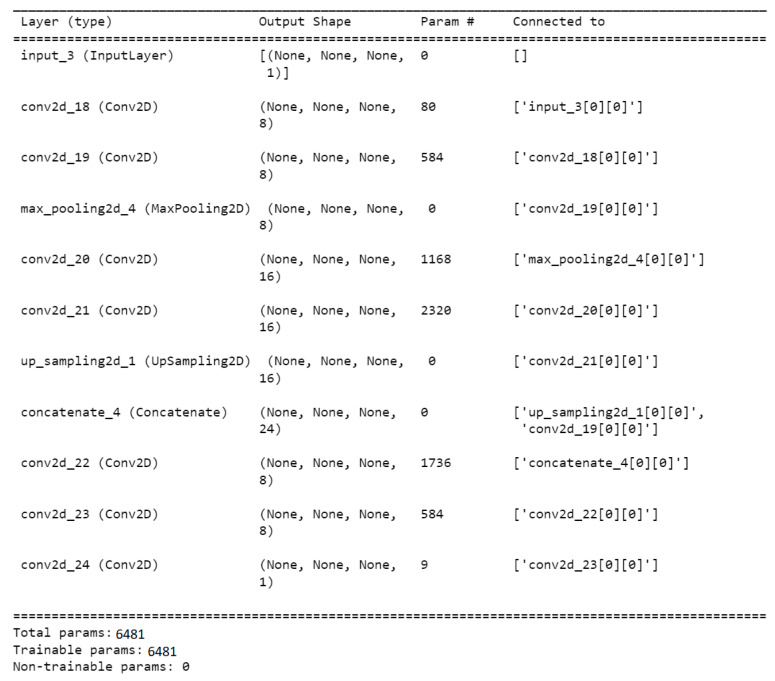
Structure of the simplified and more compact U-Net 1 architecture.

In our case, the task is relatively straightforward, requiring the model to capture a more limited range of complex features. Therefore, we make use of the simplified U-Net 0 architecture once again, maintaining a constant number of eight filters for each layer. The ReLU activation function is used for each layer, and padding is set to ‘same’ to preserve spatial dimensions. Following each convolutional layer, an identical convolutional layer is added, effectively doubling the number of feature maps. Max-pooling layers with a pool size of 2 × 2 reduce the spatial dimensions by half. The bottleneck consists of two convolutional layers with eight filters each, followed by ReLU activation functions. The expansive path involves upsampling the feature maps and concatenating them with feature maps from the contracting path. After each upsampling layer, two convolutional layers with eight filters and ReLU activation are applied. Concatenation combines the upsampled feature maps with the corresponding feature maps from the contracting path. This network, designed in this manner, consists of 6481 parameters (see Figure A2).

#### 3.1.3. Simplified U-Net 2

**Figure 6:**
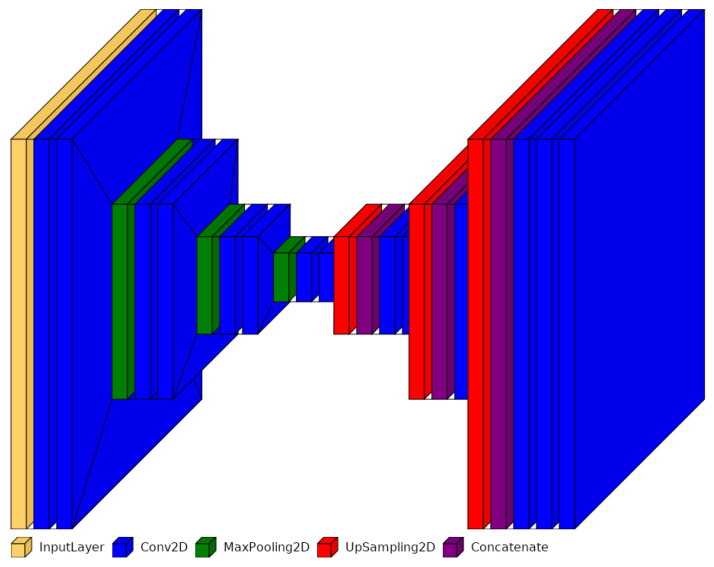
Structure of the simplified and more compact U-Net architecture.

**Figure A3:**
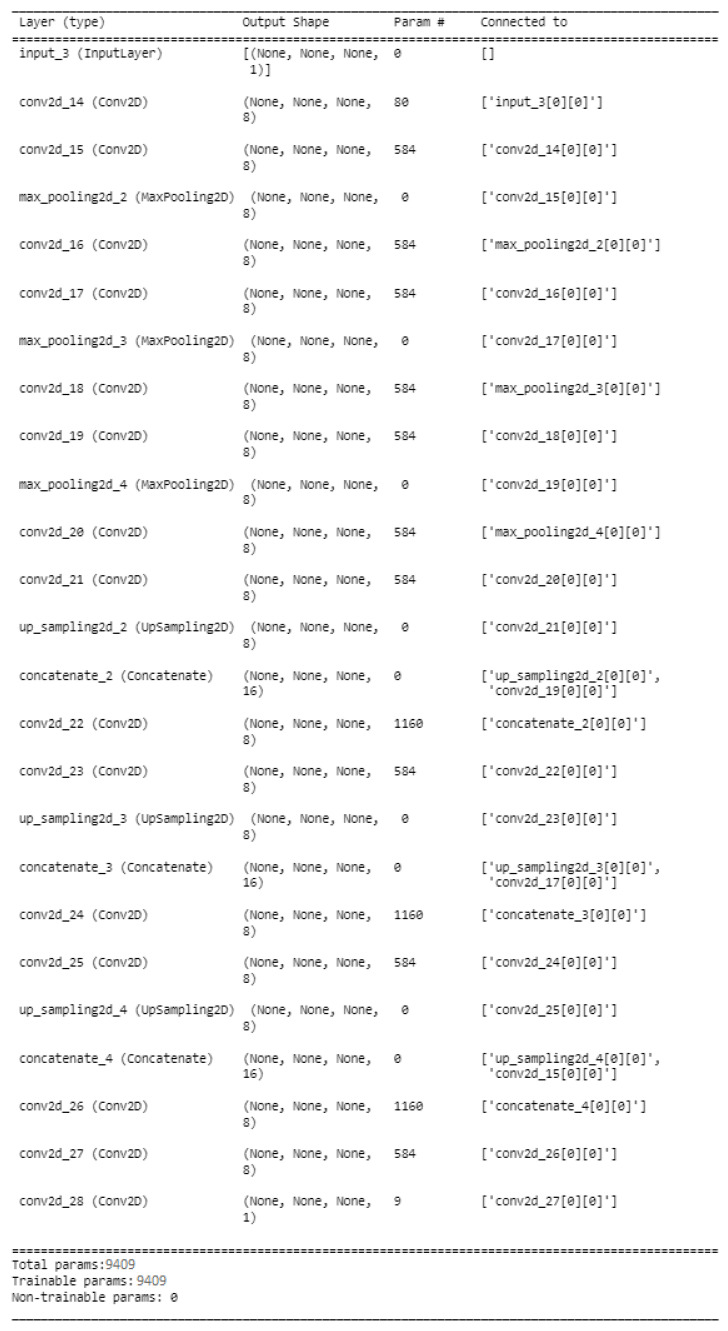
Structure of the simplified and more compact U-Net 2 architecture.

The next network is designed with increased depth. In analogy to the simplified U-Net 1, the number of filters remains constant. The contracting path comprises three convolutional segments, each followed by a max-pooling layer with a pool size of 2 × 2. Each convolutional segment consists of two identical convolution layers with eight filters activated by the ReLU activation function. Padding is set to ’same’. The bottleneck comprises two convolutional layers with eight filters each, followed by ReLU activation functions, compressing the information into a latent representation. The expansive path consists of three upsampling segments, each comprising an upsampling layer followed by two identical convolutional layers. Concatenation combines the upsampled feature maps with the corresponding feature maps from the contracting path. The network designed in this way consists of 9409 parameters (see Figure 6 and Figure A3).

## 4. Network Training Details

The U-Nets can undergo training to classify predetermined patterns using labeled training data, a process known as supervised learning. However, in the case of experimentally collected data on complex plasmas, which may consist of multiple phases, applying labels to train the networks is impractical. Therefore, the networks need to be trained with artificial data. During training, the predictions of each input are compared with their labels, and the weights are readjusted accordingly. With each iteration of this process, the accuracy of the neural network improves. An often encountered challenge is the phenomenon known as “overfitting”. In a specific example, a U-Net model is trained to transform the input into a binarized representation. In this representation, each pixel within 4.5 pixels of a particle in the input is set to 1, while every other pixel is set to 0. According to [15], the network is compiled using binary cross-entropy. Cross entropy is a mathematical concept used to measure the dissimilarity or “distance” between two probability distributions, often representing predicted and true data. In this context, it assesses how well the model’s predictions align with the true labels. Binary cross entropy is a specific form of cross entropy designed for binary classification tasks, where there are only two possible outcomes, typically denoted as classes 1 and 0.

Replicating the optical properties of the PK-4 experiment, the appearance of a particle is simulated using the open source package “deeptrack” [10]. The training set consists of synthetic images with dimensions of 512 × 512 pixels. Each image includes a minimum of 40 and a maximum of 350 particles. The particles are simulated as point scatterers, and their positions in the camera plane adhere to a normal distribution. The standard deviation for this distribution is set to 5 pixel units along the axis normal to the camera plane. Each particle is imaged using a fluorescence microscope with numerical aperture NA between 0.6 and 0.8 and illuminating laser wavelength of 532 nm. The noise is simulated as poisson noise with an signal to noise ratio in the range of 5 to a maximum of 200. Here, the range of noise is in line with the well-known Rose criterion (Rose [16] (p. 97)), which states that a signal-to-noise ratio of at least 5 is required for reliable detection [6].

Poisson noise is a basic form of uncertainty associated with the measurement of light, inherent to the quantized nature of light and the independence of photon detections. To enhance the training of the U-Net architecture, data augmentation is a powerful technique. Applying various transformations to the existing training dataset, data augmentation is used to create additional synthetic training examples. These transformations maintain the semantic content of the data while introducing diversity through variations in factors like rotation, scaling, translation, and noise. The key idea is to expose the model to a broader range of input variations, making it more robust and better at generalizing to unseen data. The image is finally normalized by rescaling it to be contained between two random numbers within (0, 1).

During training, we employ learning rate scheduling. In deep learning, using a fixed learning rate can result in divergence or slow convergence. Learning rate scheduling enables the adjustment of the learning rate throughout the training process. We initiate training with an initial learning rate of 0.001 and decrease it to 0.0001 after the first 5 epochs. This approach is designed to facilitate faster convergence in the initial training phases with a higher learning rate, while a lower learning rate later on aids in fine-tuning and stabilizing the model’s performance, bringing it closer to the optimal solution. The validation set consists of 100 images. After 100 epochs, the network achieves approximately 98% accuracy in analyzing test data.

## 5. Results on Artificial Data

**Figure 7:**
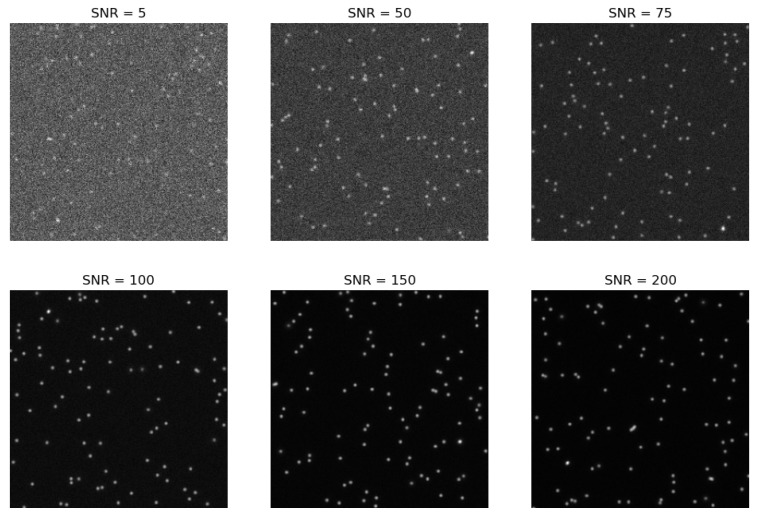
Extract of the train data. Artificial 512 × 512 pixel images, with different signal to noise ratios.

**Figure 8:**
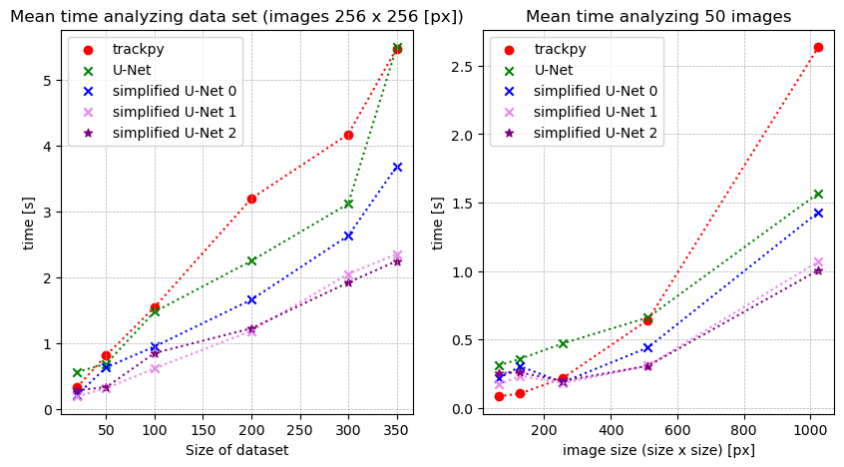
Time analyzing data sets of different sizes or image sizes at given noise level of 100.

To compare the U-Net architectures with trackpy and StarDist, we generated artificial data with a signal-to-noise ratio ranging from 5 to 200 (see Figure 7). Initially, we assess the analysis time required by the methods. We compare the time needed for datasets of various sizes, which include 256 × 256 images. Additionally, we evaluate the analysis time for images of different dimensions, such as 64 × 64, 128 × 128, 256 × 256, 512 × 512, and 1024 × 1024 pixels. It turned out that the analysis based on StarDist takes about ten times longer than using the other methods. To keep the graph clear, StarDist is not listed in Figure 8.

**Figure 9:**
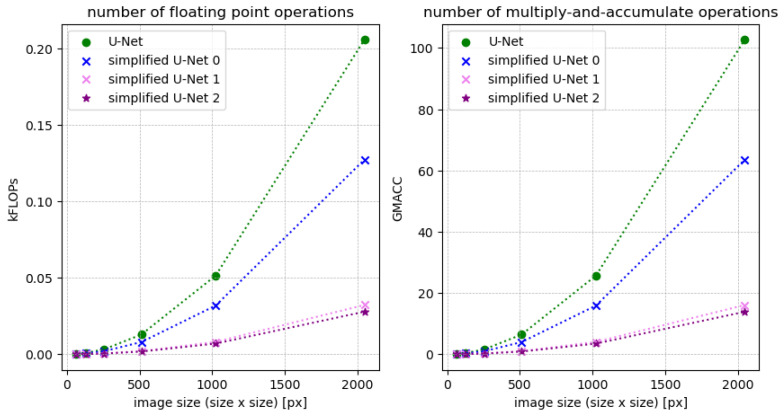
Statistical indicators for computational complexity.

Examining Figure 8, it becomes evident that the larger the dataset, the more distinct the differences in analysis times. The simplified U-Net architectures consistently exhibit the shortest analysis times, with **simplified U-Net 1** and **simplified U-Net 2** standing out. On average, analyses based on these architectures take almost half the time compared to other methods. When considering the image format, trackpy demonstrates faster performance for small images, while the U-Net architectures become more efficient as the image format increases. This suggests that operations directly applied to the image are faster for small formats, while creating masks and subsequent detection become more efficient for larger images. The computational complexity of the architectures directly influences their running speed, commonly measured by the floating-point operand FLOPs [17]. To compare the U-Net architectures, the FLOPs indicator is utilized (refer to Figure 9). FLOPs are calculated for each layer, such as convolution layers, following a specific formula:

(1) $$FLOPs=2HW\left(C_{in}K^{2}+1\right)C_{Out},$$

where *H* = height, *W* = width, $C_{in}$ = number of channels of the input feature map, *K* = kernel size and $C_{Out}$ = number of output channels [17].

The FLOPs do not depend directly on the number of parameters. Networks with the same number of parameters can have a different number of FLOPs due to different network depth or width.

In addition, we compare the architectures using the improved indicator multiply-accumulate operations (MACCs) (see Figure 9). For a conv layer with kernel size K, the number of MACCs is:

(2) $$MACC=K^{2}\;\cdot \;C_{in}\;\cdot \;H_{Out}\;\cdot \;W_{Out}\;\cdot \;C_{Out},$$

where *K* = kernel size, $C_{in}$ = number of channels of the input feature map, $H_{Out}$ = height of the output, $W_{Out}$ = width of the output and $C_{Out}$ = number of output channels [17].

Observing Figure 9, the simplified U-Net 1 and 2 stand out prominently. In direct comparison, these architectures remain remarkably compact, even with large input images. For images sized 2048×2048 pixels, the number of FLOPs is approximately one-fifth of the FLOPs of the full-size U-Net. Similarly, the number of MACCs is only one-tenth of the MACCs of the original U-Net. Consequently, these architectures are significantly less complex than the full-size model. Beyond the compactness of the architectures, their accuracy is pivotal in the analysis. Therefore, the average percentage of correctly detected particles at a given noise level is compared, considering the number ***N*** of incorrectly detected particles. Predicted particles are considered correct only if their deviation from the actual position is within 5% of the particle diameter.

**Figure 10:**
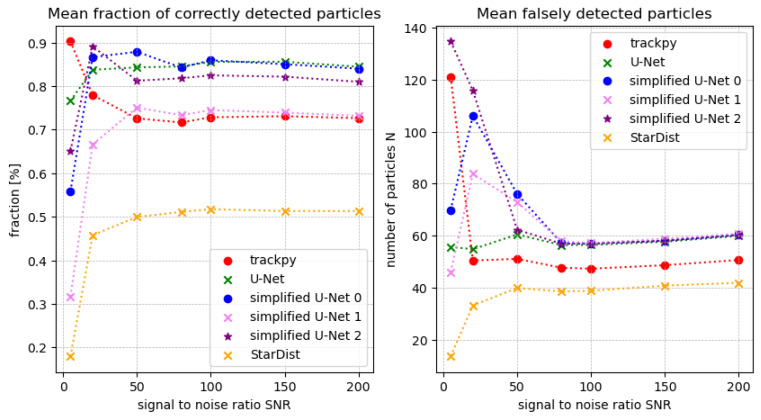
Prediction of the methods at a given noise level, taking into account misclassifications.

Figure 10 shows that trackpy [9] detects more particles at higher noise levels compared to neural networks. However, assessing the mean number ***N*** of incorrectly detected particles reveals the method’s relative inaccuracy and tendency to detect particles somewhat randomly. In strong noise conditions, the full-size **U-Net** performs well, with few misdetected particles, identifying approximately 87% of the sought particles. The simplified U-Net architectures are less accurate in significant noise. Specifically, simplified U-Net 2 behaves similarly to trackpy, with an increase in misclassifications as image noise rises. Starting from a signal-to-noise ratio greater than 50, the **simplified U-Net 0** detects particles as reliably as the full-size U-Net, with fewer false detections as noise decreases. For all methods, fewer particles are falsely detected as the noise decreases. While the number of false detections for the **simplified U-Net 1**, starting from a signal-to-noise ratio of 50, is almost identical to the number for the full-size **U-Net**, the count of correctly detected particles is slightly lower than that for the full-size **U-Net** and the **simplified U-Net 0**. Performance differences may stem from the varying complexity of these model architectures, enabling more complex ones to robustly segment and detect particles compared to simpler architectures. Notably, the **simplified U-Net 2** demonstrates commendable results, suggesting an optimal balance between efficiency and effectiveness. This is particularly relevant as data from parabolic flight campaigns typically have a signal-to-noise ratio of around 100. In contrast, the StarDist [14] model seems less suitable for detecting particles in a complex plasma. Despite having the fewest misclassifications, it only detects around half of the sought-after particles, even with a high signal-to-noise ratio (SNR). Trackpy, on the other hand, exhibits a lower percentage of correctly detected particles as noise decreases compared to the U-Net architectures, although its mean misdetected particles count is significantly lower. The observed performance differences could be attributed to the varying adaptability of the methods to changing conditions, particularly in terms of subpixel accuracy. The potential improvement in accuracy for trackpy might be achieved through adaptation to changing conditions. On the other hand, neural networks appear to work more independently in this regard, despite exhibiting a higher count of misclassified particles, especially with lower noise levels.

**Figure 11:**
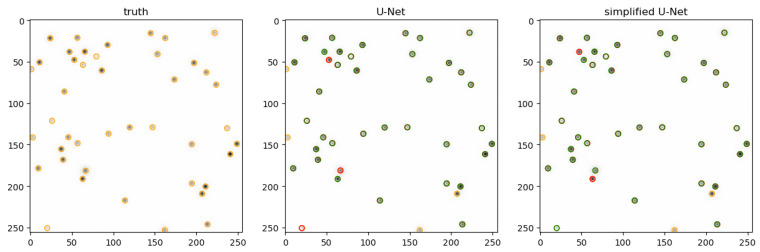
Prediction of both U-net architectures for an extract of the artificial data, where the particles marked in green were detected correctly, and the particles marked in orange were not detected. The particles marked in red show the difference between the two networks. The image shown above has been cropped and inverted for illustration.

To enhance the accuracy of neural networks, more extensive training with a diverse set of training data, including variations in signal-to-noise ratio and background noise, could be beneficial. This approach may lead to improved generalization and robustness of the networks across different conditions. An illustrative comparison of predictions between the full-size U-Net and the simplified U-Net 2 is presented in Figure 11. The visualization clearly demonstrates that certain particles are detected by the U-Net but missed by the simplified U-Net 2, and vice versa. However, with a few exceptions, it can be concluded that the two networks generally yield quite similar results.

## 6. Using the Trained Network on Experimental Data

The image data of the particles were recorded during a parabolic flight campaign by Justus Liebig University in the A310 ZERO-G aircraft of Novespace (for more details see Section 2).

**Figure 12:**
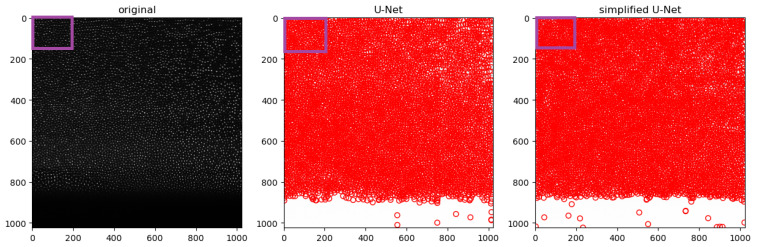
Prediction of both U-Nets for an extract of the experimental data. The image shown above should be cropped for illustration (purple rectangle).

**Figure 13:**
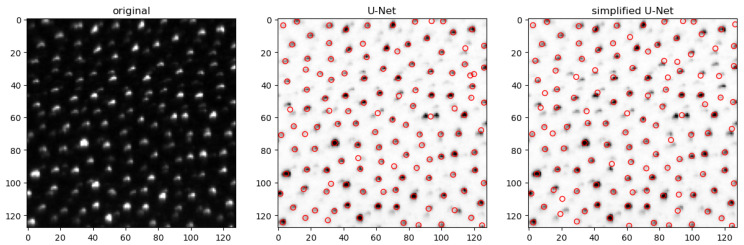
Prediction of both U-Nets for an extract of the experimental data. The image shown above has been cropped for illustration.

As this is a measurement, there are no truth data available that can be used to verify the results. The experimental data are comparatively less noisy with respect to Poisson noise. Accordingly, the simplified U-Net should also produce resilient and solid results. A look at Figure 12 shows that the images taken during parabolic flight campaigns contain a large amount of position data, which further emphasizes the need for the most efficient architecture possible. At first glance, it seems that the two U-Net architectures provide approximately similar results. On the one hand, the simplified network detects 91% of the particles that the fullsize network also detects. On the other hand, 19% of the particles detected by the simplified network are not detected by the full-size net. On closer inspection, it is noticeable that both neural networks seem to detect particles in places where apparently none should be found (see Figure 13). These ghost particles seem to occur mainly in the edge region. This may lead to inaccurate and inconclusive analyses afterwards. This problem can probably be solved with more extensive training data, which are more similar to the parabolic flight data.

## 7. Conclusions

We presented an efficient and compact U-Net version for detecting multiple particles in complex plasmas. The results also demonstrated that the compact neural network is relatively accurate, especially in low-noise data. Furthermore, the compact network exhibits relatively fast performance with large datasets or larger-format images. It is worth emphasizing that the compact U-Net is suitable for small single-board computers with limited resources due to its low runtime and memory requirements. Accordingly, there are possible applications for future parabolic flight campaigns to perform initial analyses during flight. Compared directly to U-Net architectures, trackpy appears to be constrained by the system’s complexity. The method must be repeatedly adapted to the different noise levels in order to achieve reliable results. Essentially, the method’s parameters are crucial for the analysis accuracy, making them a potential source of error. Accordingly, the settings are a potentially large source of error. On the one hand, the development with deep learning has shown that these limitations can be largely overcome. A major advantage of the machine learning approach for particle tracking is that simulated data can often be used to train the networks. In addition, no presettings have to be made. Thus, the trained networks can be used universally. However, the networks detected ghost particles in the experimental data, potentially leading to inaccurate follow-up analyses. This issue should be considered in future developments, and efforts should be made to correct the error.

In view of further developments, the network could be evolved, corresponding to recent studies [11,15,18], to trace the particles and reconstruct their three-dimensional positions or analyze potential string formations [19].

## Data Availability

The data presented in this study are available on request from the author.
